# Potentiation of sensory responses in ventrobasal thalamus *in vivo* via selective modulation of mGlu1 receptors with a positive allosteric modulator

**DOI:** 10.1016/j.neuropharm.2011.11.015

**Published:** 2012-03

**Authors:** T.E. Salt, H.E. Jones, I.M. Andolina, C.S. Copeland, J.T.C. Clements, F. Knoflach, A.M. Sillito

**Affiliations:** Department of Visual Neuroscience, UCL Institute of Ophthalmology, University College London, 11-43 Bath Street, London EC1V 9EL, United Kingdom

**Keywords:** mGlu1 receptors, Ro67-4853, Thalamus, Cortico-thalamic, Sensory processing, Attention

## Abstract

Metabotropic glutamate subtype 1 (mGlu1) receptor is thought to play a role in synaptic responses in thalamic relay nuclei. The aim of this study was to evaluate the positive allosteric modulator (PAM) Ro67-4853 as a tool to modulate thalamic mGlu1 receptors on single thalamic neurones *in vivo*. Ro67-4853, applied by iontophoresis onto ventrobasal thalamus neurones of urethane-anaesthetised rats, selectively enhanced responses to the agonist (S)-3,5-dihydroxy-phenylglycine (DHPG), an effect consistent with mGlu1 potentiation. The PAM was also able to enhance maintained responses to 10 Hz trains of sensory stimulation of the vibrissae, but had little effect on responses to single sensory stimuli. Thus Ro67-4853 appears to be a highly selective tool that can be useful in investigating how mGlu1 receptor potentiation can alter neural processing *in vivo*. Our results show the importance of mGlu1 in sensory processing and attention mechanisms at the thalamic level and suggest that positive modulation of mGlu1 receptors might be a useful mechanism for enhancing cognitive and attentional processes.

## Highlights

► We the studied effects of Ro67-4853, an mGlu1 PAM, on single thalamic cells *in vivo*. ► Ro67-4853 selectively potentiated responses to the agonist DHPG. ► Ro67-4853 potentiated sensory synaptic responses to repetitive vibrissa stimulation. ► Ro67-4853 is a selective tool to potentiate mGlu1-mediated neural processing *in vivo*. ► Positive modulation of mGlu1 may be a useful means to enhance cognition and attention.

## 1 Introduction

It is now well-established that metabotropic glutamate (mGlu) receptors participate in synaptic transmission and modulation throughout the central nervous system (Niswender and Conn, 2010). The eight known mGlu receptors can be placed into three groups on the basis of sequence homology, pharmacology and intracellular transduction mechanisms (Nakanishi, 1992; Niswender and Conn, 2010). The Group I (mGlu1 and mGlu5) receptors have a predominantly post-synaptic location where they can couple to inositol phosphate metabolism and enhance post-synaptic excitability via changes in K^+^ conductances and/or modulation of ionotropic glutamate receptors such as the N-methyl-d-aspartate (NMDA) receptor. Both mGlu1 and mGlu5 receptors are activated by the agonist (S)-3,5-dihydroxy-phenylglycine (DHPG) (Schoepp et al., 1994; Schoepp et al., 1999), thus making the selective activation of either of these receptors difficult. However, positive allosteric modulators (PAMs) that are selective for either mGlu1 or mGlu5 have more recently been developed and characterised (Niswender and Conn, 2010; Nicoletti et al., 2011). For example Ro67-4853 has been shown to potentiate mGlu1 receptor responses in a number of *in vitro* systems (Knoflach et al., 2001). Thus such a compound would be ideally suited to potentiate synaptic responses that have an mGlu1 involvement whist having little direct effect in the absence of physiological stimulation of the receptor. This would therefore be a more selective and specific way of enhancing or activating mGlu1 receptors in a neural circuit than by the application of an agonist such as DHPG. Furthermore, Ro67-4853 might be expected to reveal specific mGlu1-mediated effects on neural circuit function in a situation where these receptors are located in distinct synaptic locations.

Previous work from this laboratory has shown that mGlu1 and mGlu5 receptor activation depolarises and excites thalamic relay cells and contributes to thalamic synaptic responses *in vivo* and *in vitro* (Salt and Turner, 1998; Salt and Binns, 2000; Turner and Salt, 2000). In particular, working in various thalamic relay nuclei, we and others have shown using selective antagonists that mGlu1 receptors are associated with the corticofugal input onto thalamic relay cells (Turner and Salt, 2000; Rivadulla et al., 2002; Reichova and Sherman, 2004). This is consistent with neuroanatomical data that reveals mGlu1 receptor localisation on the dendrites of thalamic relay cells beneath or close to terminals that appear to be of cortical origin (Martin et al., 1992; Godwin et al., 1996; Vidnyanszky et al., 1996). Thus a selective potentiator of mGlu1 receptors could be a useful experimental tool to identify mGlu1 receptor function. Furthermore, such an agent might be useful as selective potentiator of activity either experimentally or therapeutically in neural pathways that utilise mGlu1.

The aim of this study was firstly to evaluate the potential of the positive allosteric modulator (PAM) of mGlu1, Ro67-4853 (Knoflach et al., 2001), to modulate mGlu1 responses *in vivo* in the thalamus at the single neurone level. Secondly, to investigate whether Ro67-4853 could affect the responses of thalamic relay cells to sensory stimuli of different types to see whether mGlu1 receptors are recruited into synaptic responses under different stimulus conditions. This is important in the understanding of the contribution of cortico-thalamic modulatory input to sensory responses and in terms of understanding how mGlu1-receptor PAMs might affect sensory processing. In order to achieve these aims we have carried out single neurone recordings and iontophoretic application of glutamatergic agents in the rat ventrobasal thalamus *in vivo*.

The results of this study show that Ro67-4853 can potentiate mGlu1-receptor-mediated responses to the agonist DHPG, and that this appears to be a selective effect. Furthermore, Ro67-4853 was found to potentiate responses of ventrobasal thalamus neurones to somatosensory stimulation in a stimulus-specific manner, consistent with a role for mGlu1 receptors in sensory processing in the thalamus.

## 2 Methods

Experiments were carried out as detailed previously in male adult Wistar rats (270–400 g) anaesthetised with urethane (1.2 g/kg, I.P.) (Salt, 1987; Salt and Binns, 2000). All procedures were approved by the Home Office (UK) and were in accordance with the Animals (Scientific Procedures) Act 1986. Electroencephalogram and electrocardiogram were recorded throughout and anaesthesia was maintained by additional I.P. administration of urethane as required. Single neurone recordings were made from ventrobasal thalamus neurones using the centre barrel (filled with 4 M NaCl) of seven-barrel glass electrodes, and substances under investigation were applied in the recording location from the six outer barrels of the electrode using the iontophoretic technique with a Neurophore BH2 system. Each of the outer barrels contained a selection from one of the following substances: NMDA (50 mM, pH 8.0 in 150 mM NaCl), LY367385 (100 mM in water, pH 8.0), DHPG (50 mM in 150 mM NaCl, pH 3.5), Ro67-4853 (2 mM in 10% DMSO in 150 mM NaCl, pH 7.5), Vehicle control (10%DMSO in 150 mM NaCl, pH 7.5), Pontamine Sky Blue dye (2% in 1 M NaCl), 1 M NaCl. DHPG was ejected as a cation, all other substances were ejected as anions. Agents were prevented from diffusing from the iontophoresis barrel by applying a retaining current (5–15 nA) of opposite polarity to the ejection current. Automatic current balancing was routinely performed through the 1 M NaCl barrel, although this was also switched off in a small number of experiments in order to verify that results were similar under both types of iontophoretic procedure.

Two types of experimental protocol were followed. Firstly, in order to evaluate the effectiveness and specificity of Ro67-4853 when applied via iontophoresis, the agonists DHPG and NMDA were applied alternately at regular intervals (typically 180 s) in order to evoke consistent sub-maximal increases in action potential firing rate, as described previously (Salt and Binns, 2000). When stable responses to the agonists were achieved, Ro67-4853 was co-applied (typically for 3–9 min) and changes in the responses to the agonists were noted. Application of Ro67-4853 was then terminated and responses to agonists continued to be recorded. Secondly, in order to evaluate the effect of the PAM on sensory input, deflection of facial vibrissae was performed using an electronically-gated air jet (10–20 ms duration) directed at a single vibrissa (Salt, 1987). Such stimuli were presented alternately as single stimuli and in trains of stimuli (10 Hz, 1 s duration), separated by 4–5 s. When stable responses to vibrissa deflection were achieved, Ro67-4853 was co-applied (typically for 3–9 min) and changes in the responses to sensory stimulation were noted. This enabled a direct comparison of the effects of R067-4853 on the two modes of sensory stimulation. Application of Ro67-4853 was then terminated and responses to sensory stimulation continued to be recorded.

Action potential signals were amplified using an Axoprobe 1A amplifier, gated, timed and recorded via a CED1401 interface hosted on a computer running Spike2 software (Cambridge Electronic Design, UK). Data were analysed by plotting peristimulus time histograms (PSTHs) from these recordings and counting the spikes evoked by either sensory stimulation or agonist ejection. Response magnitudes were calculated by counting action potential spikes evoked by each agonist or sensory stimulus under control conditions, during application of the PAM, and after the end of the PAM application during the washout (recovery) phase. Responses in the presence of PAM were routinely expressed as a percentage of the pre-PAM ejection control response. Non-parametric statistics (Kruskal–Wallis test and Wilcoxon matched-pairs test) were used to test for significant differences between control and PAM conditions.

## 3 Results

### 3.1 Effects of Ro67-4853 on responses of thalamic neurones to iontophoretically applied DHPG and NMDA

**Fig. 1:**
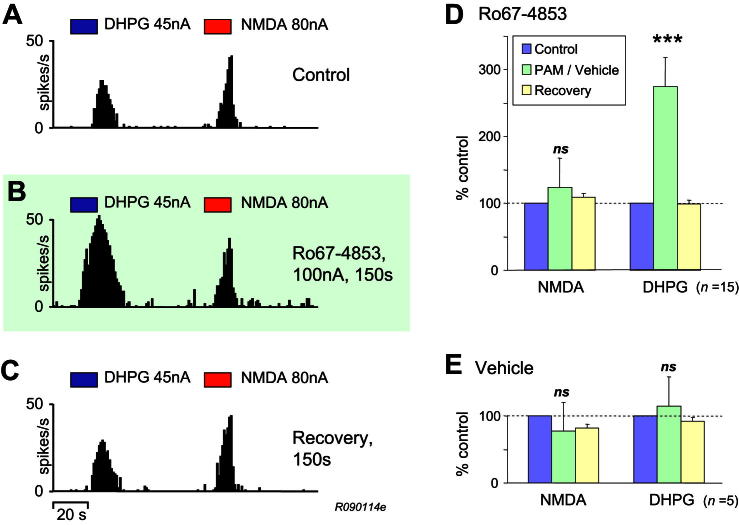
Selective potentiation of DHPG responses by Ro67-4853. A–C: Records are counts of action potentials (in 1 s epochs) recorded from a single neurone in response to application of either DHPG or NMDA, as indicated by the marker bars above the records. A – Control responses. B – Responses during the co-application of Ro67-4853 (that had commenced 150 s previously). C – Recovery (washout) from the Ro67-4853 effect. Record commenced 150 s after the end of the PAM application. D & E: Overall effect of Ro67-4853 or Vehicle on agonist responses. Bars represent mean % change from control (100%) of responses to either NMDA or DHPG during application of the PAM or Vehicle, and recovery of agonist responses after termination of the PAM or Vehicle application. D – Ro67-4853 produced a significant selective potentiation of DHPG responses, with little effect on NMDA responses. Data from 15 neurones. E – Passing iontophoretic current through Vehicle-containing barrel had no significant effect on responses to either NMDA or DHPG. Data from 5 neurones that were also tested with Ro67-4853 and included in A. ***P < 0.001; ns not significant.

In agreement with our previous findings, VB neurones under our experimental conditions were almost quiescent, and iontophoretic applications of DHPG or NMDA produced increases in action potential firing of thalamic neurones (see Fig. 1A) as reported previously by ourselves (Salt et al., 1999; Salt and Binns, 2000). When Ro67-4853 was co-applied (100–200 nA, mean = 115 ± 11 nA), the PAM enhanced responses to DHPG with relatively little effect on responses to NMDA (Fig. 1B). Ro67-4853 was applied to 15 neurones that were excited by alternate applications of DHPG and NMDA, and overall a selective enhancement of DHPG responses to 275% (±35%, *P* < 0.001) of control values was seen whereas responses to NMDA were not significantly different to control (124 ± 11%) (Fig. 1D). In 5 of these neurones a vehicle control was also performed where the same iontophoretic current was passed through a barrel containing the vehicle used for Ro67-4853: in these cases no significant overall potentiation of either agonist was seen (Fig. 1E). In order to exclude the possibility that there was some interaction between NMDA and DHPG responses a further 9 neurones were recorded where DHPG was regularly ejected without alternating NMDA applications, and in these experiments application of Ro67-4853 was found to have the same potentiating effect on DHPG responses as before (246 ± 44% of control, *n* = 9, *P* < 0.005).

### 3.2 Effects of Ro67-4853 on responses of thalamic neurones to somatosensory stimuli

**Fig. 2:**
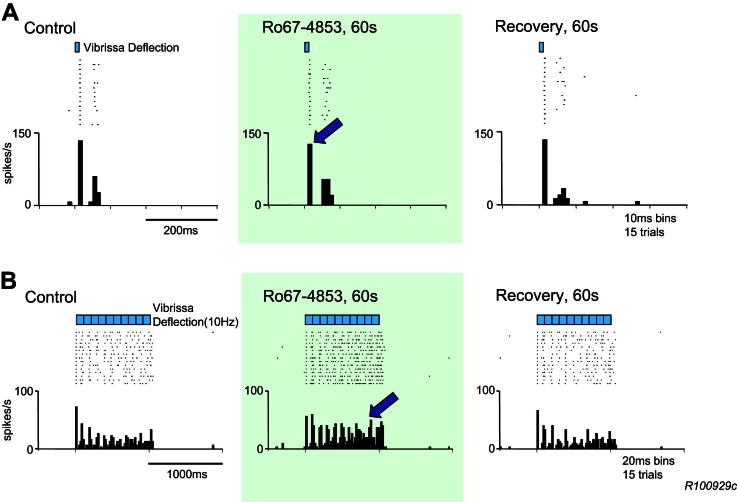
Potentiation of sensory responses of VB thalamus neurones by Ro67-4853. Raster displays and peristimulus time histograms of responses of a VB thalamus neurone to 15 trials of either a single stimulus (A) or train of stimuli (B) to a vibrissa, presented at the times marked by the bars above the raster displays. A – Ro67-4853 did not potentiate responses to single stimuli (arrow). Note also that following the initial action potential spike response, there are further spike responses that are attributable to relaxation of GABAergic inhibition arising from the thalamic reticular nucleus. B – Ro67-4853 potentiated the later response component to the train stimulus (arrow), but not the initial component.

**Fig. 3:**
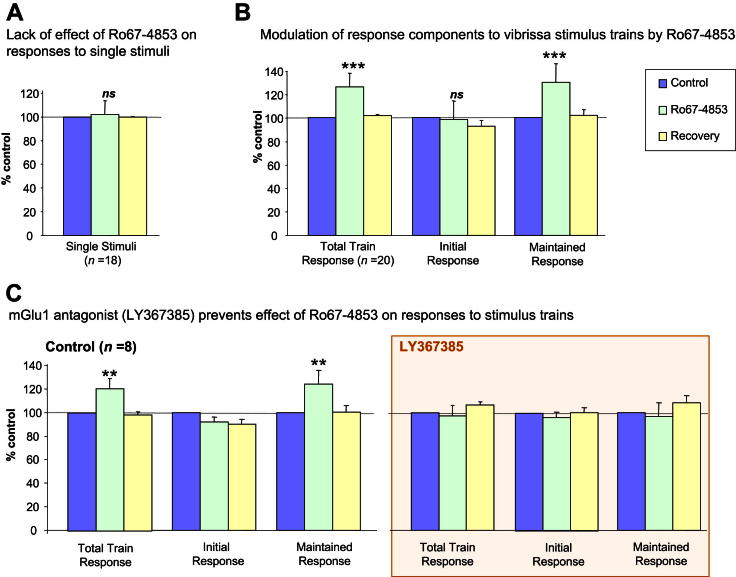
Overall effect of Ro67-4853 on responses to single stimuli or trains of stimuli directed at vibrissae. Bars represent mean % change from control (100%) of responses to vibrissa stimulation during application of the PAM and recovery of responses after termination of the PAM application. A – Responses of 18 neurones to single stimuli were not affected by the PAM. B – Responses of 20 neurones to trains of vibrissa stimulation were potentiated by PAM application. When responses were segregated into the initial response component (to the first stimulus in the train) and the remaining maintained response component, it was the latter that was potentiated by the PAM. C – Responses of 8 of the neurones shown in B showing the effect of PAM application on maintained sensory responses under control conditions, and during co-application of the mGlu1 antagonist LY367385 (box). Note that LY367385 prevented the potentiation of responses by the PAM. ***P < 0.001; **P < 0.01; *P < 0.05; ns not significant.

Vibrissal stimulation delivered as single deflections (10–20 ms) typically produced responses of one to three action potentials at a latency of 12–30 ms, as described previously (Salt, 1987). When stimuli were presented in 10 Hz trains, the responses to the first stimulus in the train were similar to those seen in response to single stimuli, but the responses to subsequent stimuli in the train were usually reduced (Fig. 2B). Iontophoretic application of Ro67-4853 did not significantly alter the responses to single stimuli (102% ± 3% of control, *n* = 18), but it did potentiate responses to trains of stimuli, as shown in the example in Fig. 2 and summarised in Fig. 3A and B. Overall, responses of 20 neurones to trains of stimuli were enhanced to 125% (±6%) of control responses (*P* < 0.001) (Fig. 3B). Furthermore, analysis of the responses to trains of deflections revealed that latter components of the train response (response to last nine stimuli in the train) were potentiated by the PAM (131% ± 7%, *P* < 0.001), whereas the initial component (response to the first stimulus of the train) remained unaffected (99% ± 4% of control) (Fig. 3B).

In order to verify that the potentiations by Ro67-4853 of responses to trains of vibrissa stimulation were indeed mediated via mGlu1 receptors, we proceeded to eject a selective mGlu1 receptor antagonist, LY367385 (Clark et al., 1997; Salt and Binns, 2000), continuously (40 nA) on 8 of the 20 neurones after recovery from the Ro67-4853 application. In agreement with our previous findings (Salt and Turner, 1998), LY367385 had no significant effect on sensory responses. Subsequent application of Ro67-4853 during the LY367385 ejection was without effect on the maintained vibrissal responses to trains of stimuli (97% ± 12% of control). Furthermore, after termination of the LY367385 ejection, when the stimulation and Ro67-4853 application protocol was repeated, Ro67-4853 was found to potentiate responses again (136% ± 20% of control). These data are summarised in Fig. 3C.

## 4 Discussion

The data reported here represent, to our knowledge, the first *in vivo* characterisation of Ro67-4853 at the single neurone level. Our findings are in accord with previous *in vitro* work (Knoflach et al., 2001), and show that Ro67-4853 can act as a selective positive modulator of mGlu1 receptor-mediated responses in the thalamus *in vivo*. Furthermore, this property can be exploited to selectively enhance mGlu1-mediated synaptic response components.

Previous work from this laboratory has shown that VB thalamus neurones can be excited by activation of Group I mGlu receptors. Furthermore, we have shown that the post-synaptic excitation evoked by agonists such as DHPG is mediated predominantly via the mGlu1 receptor subtype rather than the mGlu5 subtype (Salt et al., 1999; Salt and Binns, 2000). This is consistent with the high levels of expression of this receptor on neurones in this part of the brain (Martin et al., 1992; Shigemoto et al., 1992; Neto et al., 2000; Ngomba et al., 2011a). In the present study, we have shown that Ro67-4853 can potentiate responses to DHPG whilst having relatively little overall effect on responses to NMDA. This indicates that positive allosteric modulation of mGlu1 receptors can be achieved using Ro67-4853 *in vivo*. It is noteworthy that we have previously found that NMDA receptor responses can be potentiated by activation of mGlu1 receptors using DHPG *in vivo* (Salt and Binns, 2000). Our present finding that NMDA responses were not affected by Ro67-4853 application would suggest that there is little tonic activation of mGlu1 receptors under our experimental conditions.

In the present study we also investigated the effects of the mGlu1 PAM on responses of VB neurones to vibrissa stimulation. Our previous work has found that vibrissal responses are mediated by ionotropic glutamate receptors of both the non-NMDA and NMDA type (Salt, 1987; Salt and Eaton, 1991) with little apparent involvement of mGlu receptors, as indicated by the lack of effect of various competitive mGlu receptor antagonists (Eaton et al., 1993; Salt and Eaton, 1994), including the mGlu1-selective antagonist LY367385 (Salt and Turner, 1998). Interestingly, we found that Ro67-4853 could nevertheless enhance components of vibrissal responses under certain conditions: in particular we found that responses to repetitive vibrissa stimulation were enhanced by the PAM whereas responses single stimuli or to the initial component of a repetitive stimulus were unaffected. This, together with the lack of effect of mGlu antagonists on sensory responses (see above), suggests that an mGlu1-receptor component is present but sub-threshold under our experimental conditions, and that this can be selectively enhanced by the PAM. There is considerable anatomical and physiological evidence showing that mGlu1 receptors are post-synaptic to corticofugal afferents terminating on the dendrites of thalamic relay cells but are not post-synaptic to sensory afferent terminals (Martin et al., 1992; Godwin et al., 1996; Vidnyanszky et al., 1996; Guillery and Sherman, 2002). This, together with our current findings with Ro67-4853, suggests that application of the PAM results in a selective enhancement of the cortical input to VB cells in a stimulus-specific manner. Furthermore, the finding that the PAM enhances the latter components of sensory responses is consistent with such a view, as it might be expected that a facilitatory cortical feedback operating via a synaptic mGlu receptor would show a requirement for repeated activation and a relatively slow time course.

It has been suggested that the modulatory cortico-thalamic pathways that project from Layer 6 of the cortex to the thalamic relay nuclei may play an important role in governing sensory processing in the thalamus and attentional mechanisms (Guillery and Sherman, 2002; Sherman and Guillery, 2002). Our finding that it is possible to enhance presumed cortico-thalamic inputs to the thalamus with Ro67-4853 has important implications in this sphere. Firstly, our results indicate that it is possible to pharmacologically enhance the cortico-thalamic contribution to sensory processing in a specific manner so as to enhance thalamic sensory responses. This will in turn be fed to the cortex and result in increased cortical responses that will in turn be projected back down to the thalamus. Secondly, our results indicate that drugs that have mGlu1 receptor PAM activity could be a powerful means of modulating central sensory processing and they could have a function as cognitive enhancers. In this respect it is also of interest to note that a different mGlu1 PAM has been recently shown to affect spike-and-wave discharges in a genetic model of absence epilepsy, an effect that could be due to modulation of cortico-thalamic feedback projections (Ngomba et al., 2011a,b).

In conclusion, we have shown that Ro67-4853 appears to be a highly selective tool that should be useful in investigating how mGlu1 receptor potentiation can alter neural processing *in vivo*. This will be important in terms of developing a proof of concept that mGlu1 receptor modulation could provide useful central nervous system effects. Furthermore, our results underline the importance of mGlu1 in sensory processing and attention mechanisms at the thalamic level and suggest that positive modulation of mGlu1 receptors might be a useful mechanism for enhancing cognitive and attentional processes.

## References

[bib1] Clark B.P., Baker S.R. (1997). (+)-2-Methyl-4-carboxyphenylglycine (LY367385) selectively antagonises metabotropic glutamate mGluR1 receptors. Bioorganic & Medicinal Chemistry Letters.

[bib2] Eaton S.A., Birse E.F. (1993). Mediation of thalamic sensory responses in vivo by ACPD-activated excitatory amino acid receptors. European Journal of Neuroscience.

[bib3] Godwin D.W., Van Horn S.C. (1996). Ultrastructural localization suggests that retinal and cortical inputs access different metabotropic glutamate receptors in the lateral geniculate nucleus. Journal of Neuroscience.

[bib4] Guillery R.W., Sherman S.M. (2002). Thalamic relay functions and their role in corticocortical communication: generalizations from the visual system. Neuron.

[bib5] Knoflach F., Mutel V. (2001). Positive allosteric modulators of metabotropic glutamate 1 receptor: characterization, mechanism of action, and binding site. Proceedings of the National Academy of Sciences of the United States of America.

[bib6] Martin L.J., Blackstone C.D. (1992). Cellular localization of a metabotropic glutamate receptor in rat brain. Neuron.

[bib7] Nakanishi S. (1992). Molecular diversity of glutamate receptors and implications for brain function. Science.

[bib8] Neto F.L., Schadrack J. (2000). Differential distribution of metabotropic glutamate receptor subtype mRNAs in the thalamus of the rat. Brain Research.

[bib9] Ngomba R.T., Santolini I. (2011). Protective role for type-1 metabotropic glutamate receptors against spike and wave discharges in the WAG/Rij rat model of absence epilepsy. Neuropharmacology.

[bib10] Ngomba R.T., Santolini I. (2011). Metabotropic glutamate receptors in the thalamocortical network: strategic targets for the treatment of absence epilepsy. Epilepsia.

[bib11] Nicoletti F., Bockaert J. (2011). Metabotropic glutamate receptors: from the workbench to the bedside. Neuropharmacology.

[bib12] Niswender C.M., Conn P.J. (2010). Metabotropic glutamate receptors: physiology, pharmacology, and disease. Annual Review of Pharmacology and Toxicology.

[bib13] Reichova I., Sherman S.M. (2004). Somatosensory cortico-thalamic projections: distinguishing drivers from modulators. Journal of Neurophysiology.

[bib14] Rivadulla C., Martinez L.M. (2002). Completing the corticofugal loop: a visual role for the corticogeniculate type 1 metabotropic glutamate receptor. Journal of Neuroscience.

[bib15] Salt T.E., Binns K.E. (2000). Contributions of mGlu1 and mGlu5 receptors to interactions with N-methyl-d-aspartate receptor-mediated responses and nociceptive sensory responses of rat thalamic neurones. Neuroscience.

[bib16] Salt T.E., Eaton S.A. (1991). Sensory excitatory postsynaptic potentials mediated by NMDA and non-NMDA receptors in the thalamus *in vivo*. European Journal of Neuroscience.

[bib17] Salt T.E., Eaton S.A. (1994). The function of metabotropic excitatory amino acid receptors in synaptic transmission in the thalamus: studies with novel phenylglycine antagonists. Neurochemistry International.

[bib18] Salt T.E., Turner J.P. (1998). Reduction of sensory and metabotropic glutamate receptor responses in the thalamus by the novel mGluR1selective antagonist (S) 2-methyl-4-carboxy-phenylglycine. Neuroscience.

[bib19] Salt T.E., Turner J.P. (1999). Evaluation of agonists and antagonists acting at group I metabotropic glutamate receptors in the thalamus *in vivo*. Neuropharmacology.

[bib20] Salt T.E. (1987). Excitatory amino acid receptors and synaptic transmission in the rat ventrobasal thalamus. Journal of Physiology.

[bib21] Schoepp D.D., Goldsworthy J. (1994). 3,5-dihydroxyphenylglycine is a highly selective agonist for phosphoinositide-linked metabotropic glutamate receptors in the rat hippocampus. Journal of Neurochemistry.

[bib22] Schoepp D.D., Jane D.E. (1999). Pharmacological agents acting at subtypes of metabotropic glutamate receptors. Neuropharmacology.

[bib23] Sherman S.M., Guillery R.W. (2002). The role of the thalamus in the flow of information to the cortex. Philosophical Transactions of the Royal Society London B: Biological Sciences.

[bib24] Shigemoto R., Nakanishi S. (1992). Distribution of the mRNA for a metabotropic glutamate receptor (mGluR1) in the central nervous system: an in situ hybridization study in adult and developing rat. Journal of Comparative Neurology.

[bib25] Turner J.P., Salt T.E. (2000). Synaptic activation of the group I metabotropic glutamate receptor mGlu1 on the thalamocortical neurones of the rat dorsal lateral geniculate nucleus *in vitro*. Neuroscience.

[bib26] Vidnyanszky Z., Görcs T.J. (1996). Immunohistochemical visualization of the mGluR1a metabotropic glutamate receptor at synapses of cortico-thalamic terminals originating from area 17 of the rat. European Journal of Neuroscience.

